# Clinical outcomes of Sacubitril/Valsartan in patients with acute heart failure: A multi-institution study

**DOI:** 10.1016/j.eclinm.2021.101149

**Published:** 2021-10-08

**Authors:** Dong-Yi Chen, Chun-Chi Chen, Chi-Nan Tseng, Shao-Wei Chen, Shang-Hung Chang, Wen-Kuan Huang, Ming-Shien Wen, Ming-Jer Hsieh, I-Chang Hsieh

**Affiliations:** a Division of Cardiology, Department of Internal Medicine, Chang Gung Memorial Hospital Linkou, Chang Gung University College of Medicine, Taoyuan, Taiwan; b Department of Thoracic and Cardiovascular Surgery, Chang Gung Memorial Hospital Linkou, Chang Gung University College of Medicine, Taoyuan, Taiwan; c Center for Big Data Analytics and Statistics, Chang Gung Memorial Hospital, Linkou, Taiwan; d Division of Hematology/Oncology, Chang Gung Memorial Hospital Linkou, Chang Gung University College of Medicine, Taoyuan, Taiwan; e Cardio-Oncology Program, Chang Gung Memorial Hospital Linkou, Taoyuan, Taiwan

**Keywords:** Sacubitril/Valsartan, Hear failure, HFrEF, Angiotensin receptor neprilysin inhibitor (ARNI)

## Abstract

**Background:**

The effectiveness and safety of initiating sacubitril/valsartan therapy among patients who are hospitalized for acute heart failure (HF) is unclear.

**Methods:**

A cohort of 3736 patients with HF with reduced ejection fraction (HFrEF) hospitalized for acute HF was identified from Chang Gung Research Database between January 1, 2016 and August 31, 2019. The risks of rehospitalization for HF and death associated with sacubitril/valsartan therapy compared to angiotensin-converting enzyme inhibitor (ACEI) or angiotensin II receptor blocker (ARB) therapy were evaluated. We used stabilized inverse probability of treatment weighting to balance the baseline covariates. The risks of fatal and non-fatal outcomes between the groups were compared using a Cox proportional hazard model and Fine and Gray subdistribution hazard model, respectively.

**Findings:**

The composite of rehospitalization for HF and death occurred in 22.9% of the patients in the sacubitril/valsartan group compared to 32.6% in the ACEI/ARB group (hazard ratio [HR] 0.71, 95% confidence interval [CI] 0.52–0.97) after a mean follow-up period of 11.8 months. The sacubitril/valsartan group had a lower risk of rehospitalization for HF (subdistribution HR 0.83, 95% CI 0.74–0.92) and all-cause death (HR 0.51, 95% CI 0.27–0.94). There were no significant differences in the rates of worsening renal function or severe hyperkalemia between the two groups.

**Interpretation:**

In real-world practice, initiating sacubitril/valsartan therapy among patients with HFrEF who were hospitalized for acute HF was associated with a lower rate of rehospitalization for HF and death compared with ACEI/ARB therapy.

**Funding:**

This study was supported by Novartis Pharmaceuticals.

**Research in context**

**Evidence before this study**

The PIONEER-HF trial demonstrated that, compared with angiotensin-converting enzyme inhibitor (ACEI) enalapril, initiating sacubitril/valsartan treatment among patients with hospitalization for acute heart failure (HF) resulted in a lower composite of rehospitalization for HF and cardiovascular death over a short period of 8 weeks. However, the effectiveness and safety outcomes of initiating sacubitril/valsartan among patients hospitalized for acute HF in longer follow-up duration remains unclear. Furthermore, no previous study has compared the clinical outcomes of sacubitril/valsartan with ARB treatment in patients with acute HF.

**Added value of this study**

Initiating sacubitril/valsartan therapy among patients with HF with reduced ejection fraction (HFrEF) who were hospitalized for acute HF was associated with a lower rate of rehospitalization for HF and death compared with ACEI/ angiotensin II receptor blocker (ARB) therapy after a mean follow-up period of 11.8 months. There were no significant differences in the cumulative incidence rates of rehospitalization for HF or death among the different types of ACEIs or ARBs. This cohort study included a wider range of acute HF patients of PIONEER-HF non-eligible populations, such as patients with eGFR <30 mL/min/1.73 m^2^, moderate or severe mitral regurgitation, as well as patients with acute MI or receiving PCI during the index hospitalization. This study adds to the knowledge regarding evidence for the in-hospital initiation of sacubitril/valsartan compared with ACEI/ARB treatment in patients hospitalized for acute HF.

**Implications of all the available evidence**

Among patients who are hospitalized for acute HF, initiating sacubitril/valsartan therapy had lower risk of rehospitalization for HF and death. These findings may help to guide clinicians with regards to the optimal therapy for patients with acute HF after hemodynamic stabilization.

Alt-text: Unlabelled box

## 1 Introduction

Hospitalized patients with acute heart failure (HF) are at a high risk of poor outcomes, including high inpatient mortality, frequent rehospitalizations for worsening HF and death in the vulnerable post-discharge period [1,2]. The PIONEER-HF trial (Comparison of Sacubitril/Valsartan versus Enalapril on Effect on NT-pro BNP [N-terminal pro-B type natriuretic peptide] in Patients Stabilized from an Acute HF Episode) demonstrated that initiating sacubitril/valsartan treatment in patients stabilized during hospitalization for acute HF resulted in a greater reduction in NT-pro BNP concentration compared with the angiotensin-converting enzyme inhibitor (ACEI) enalapril [3]. Further exploratory analysis showed that compared with enalapril, sacubitril/valsartan significantly reduced the composite of rehospitalization for HF and cardiovascular death over a short period of 8 weeks (hazard ratio (HR), 0.58 [95% confidence interval (CI), 0.39–0.87]) [4]. However, of note, the PIONEER-HF study was not a conventional cardiovascular outcome trial, and the primary outcome was the change in NT-pro BNP concentration. Furthermore, the exploratory analysis was limited by a short follow-up period of 8 weeks. Therefore, the more relevant clinical question of the effectiveness and safety outcomes of initiating sacubitril/valsartan among patients who are hospitalized for acute HF in longer follow-up duration remains unclear. Angiotensin receptor blockers (ARBs) are more commonly prescribed than ACEIs for patients who are hospitalized for worsening HF because of ACEI-associated cough [5]. However, no previous study has compared the clinical outcomes of sacubitril/valsartan with ARB treatment in patients with acute HF. Therefore, we conducted this retrospective cohort study to assess the effectiveness and safety associated with sacubitril/valsartan compared with ACEIs/ARBs in treating hemodynamically stabilized patients during hospitalization for acute HF.

## 2 Methods

The Institutional Review Board of Chang Gung Memorial Hospital (CGMH) approved this cohort study and waived the need for informed consent because all patient data were deidentified before analysis (IRB No. 201901843B0). This study follows the Strengthening the Reporting of Observational Studies in Epidemiology (STROBE) reporting guidelines.

### 2.1 Data Sources

This study analyzed data from the Chang Gung Research Database (CGRD), which is a multi-institutional medical records database of the CGMH system [6,7]. The CGMH system has been described previously [8]. In brief, it includes seven branches (four tertiary academic medical centers and three teaching hospitals) across Taiwan, with a total of 10,050 beds and 2.4 million hospitalizations every year. The CGRD is comprised of records of all emergency services, inpatient and outpatient visits from the CGMH system, and includes demographic data, nursing records, medical charts, pharmacy details, laboratory reports, imaging results, and discharge summaries. Disease diagnoses and procedures are recorded using International Classification of Diseases, Ninth Revision, Clinical Modification (ICD-9-CM) codes before 2016, and International Classification of Diseases, Tenth Revision, Clinical Modification (ICD-10-CM) codes after 2016. The ICD-9-CM and ICD-10-CM diagnostic codes used in this study are listed in eTable 1 in the Supplement.

### 2.2 Study population and exposure medications

**Fig. 1:**
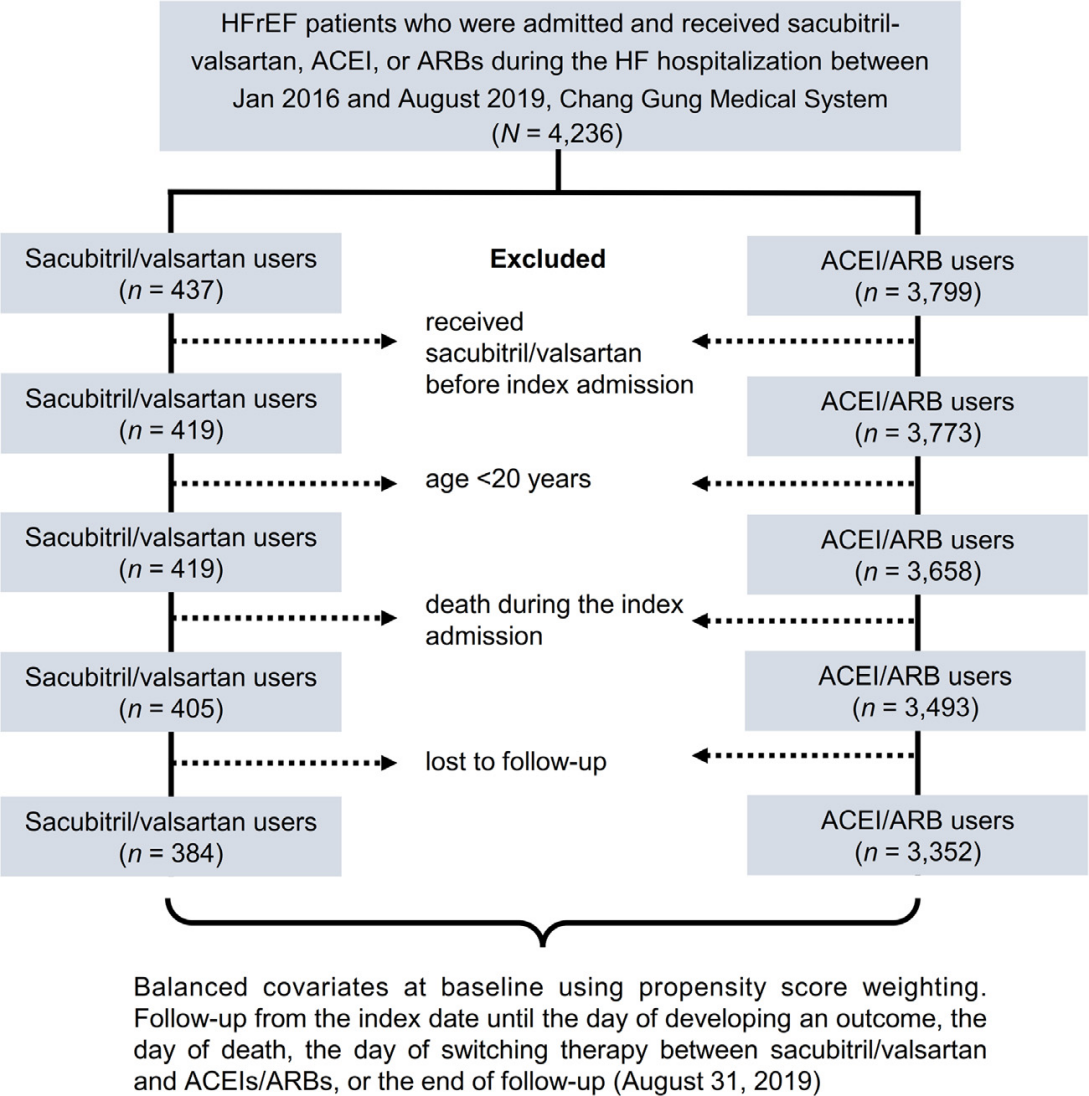
Enrollment and follow-up of the study patients. HFrEF, heart failure with reduced ejection fraction, ACEI, angiotensin-converting enzyme inhibitor, ARB, angiotensin receptor blocker.

HFrEF patients who were hospitalized due to acute HF who received sacubitril/valsartan (Anatomical Therapeutic Chemical [ATC] classification system code, C09DX04) or comparison drugs both during the hospitalization and at the date of discharge between January 1, 2016 and August 31, 2019, were identified from the CGRD. The comparison drugs were any one of the following ACEIs or ARBs: ACEIs, captopril (ATC code, C09AA01), enalapril (ATC code, C09AA02) and fosinopril (ATC code, C09AA09); ARBs, losartan (ATC code, C09CA01, C09DA01), valsartan (ATC code, C09CA03, C09DA03, C09DB01, C09DX01) and candesartan (ATC code, C09CA06, C09DA06, C09DB07). The discharge date from the index admission was defined as the index date. HFrEF was defined as a left ventricular ejection fraction (LVEF) of <40% using echocardiography information in the CGRD [9]. According to Taiwan's National Health Insurance regulations, patients with HFrEF need to have been on ACEIs or ARBs for at least 4 weeks before they can use sacubitril/valsartan. Patients were excluded if they received sacubitril/valsartan before the index admission (*n* = 44), were aged <20 years (*n* = 115), died during the index admission (*n* = 179), or were lost to follow-up (*n* = 162), which was defined as no further recorded visits in the CGMH system. After relevant exclusion, a total of 3736 patients with HFrEF who were hospitalized for acute HF were included in this study, including 384 in the sacubitril/valsartan group and 3352 in the ACEI/ARB group (Fig. 1).

### 2.3 Covariates

The covariates were demographic characteristics (age, sex, smoking and body mass index [BMI]), vital signs (heart rate, systolic and diastolic blood pressures), comorbidities, baseline echocardiography, laboratory data, concomitant medications, previous treatments for HF (including a history of implantable cardioverter–defibrillator [ICD] or cardiac resynchronization therapy [CRT]) and in-hospital events. Smoking status was recorded in the nursing care sub-database of CGRD. Information on BMI and vital signs were extracted from the medical records in the previous 3 months. Comorbidities (hypertension, diabetes, dyslipidemia, atrial fibrillation, myocardial infarction [MI], stroke, coronary artery disease, chronic obstructive pulmonary disease) were defined by the presence of two outpatient diagnoses or any one inpatient diagnosis prior to the index admission. Baseline echocardiography data in the previous 3 months were recorded, including LVEF, left ventricular (LV) end-diastolic diameter, LV end-systole diameter, left atrial diameter and mitral regurgitation severity. Laboratory data at baseline in the previous 3 months were also recorded, including B-type natriuretic peptide (BNP), blood urine nitrogen, serum creatinine, sodium, potassium and hemoglobin. Data on the use of medications in the previous 3 months were extracted, including thiazolidinedione, glucagon-like peptide-1 receptor agonists (GLP1RAs), sodium glucose cotransporter 2 inhibitors (SGLT2is), beta-blockers, mineralocorticoid antagonists, ivabradine, loop diuretics, digoxin and amiodarone. Previous treatments for HF, including ICD or CRT were also identified prior to the index date, which could be tracked back to 2001. To assess the severity of HF, we also examined HF admissions in the previous year, the frequency of HF admissions in the previous 3 years, and index hospitalization events such as hospital days, intensive care unit (ICU) days, the use of inotropic agents (including dopamine, norepinephrine, and epinephrine), intubation records, acute MI or receiving a percutaneous coronary intervention (PCI) during the index admission.

### 2.4 Outcomes

The primary effectiveness outcome was a composite outcome of rehospitalization for HF and all-cause death. Other outcomes of interest included non-fatal MI, non-fatal stroke, rehospitalization for HF, and all-cause death. The safety outcomes included worsening renal function (an increase in the serum creatinine concentration of ≥0.5 mg/dL and a decrease in the estimated glomerular filtration rate [eGFR] of ≥25%), a decline in eGFR >50% from baseline, progression to end-stage renal disease (ESRD), elevation of creatinine of ≥2.5 mg/dL, elevation of creatinine of ≥3 mg/dL, and severe hyperkalemia (potassium level >6.0 mEq/L) during follow-up. The patients were followed up from the index date (the discharge date from the index admission) until the day of developing an outcome, the day of death, the day of switching therapy between sacubitril/valsartan and ACEIs/ARBs, or the end of follow-up (August 31, 2019), whichever occurred first.

### 2.5 Statistical Analysis

Continuous variables were reported as mean ± standard deviation (SD), and categorical data as frequency (percentage). In adjustments for confounders, we created an inverse probability of treatment weighting (IPTW) cohort based on the propensity score derived from multivariable logistic regression analysis. The propensity score was calculated using the values of the covariates (detailed in Table 1) and the index date. We used a stabilized weight to mitigate the influence of outliers on the estimated propensity scores. The quality of weighting was checked using the absolute value of the standardized difference (STD) between the groups after weighting, where a value of <0.1 was considered to be a negligible difference, and a value ranging from 0.1 to 0.2 was considered to be a small difference [10,11]. Because some laboratory data were missing, we imputed the missing values using the single expectation–maximization imputation method and then created the IPTW cohort.

**Table 1:** Baseline demographics and clinical characteristics.

	Valid n	All (*n* = 3,736)	Before propensity score weighting^a^			After propensity score weighting^a^		
Variable			Sacubitril/valsartan (*n* = 384)	ACEI/ARB (*n* = 3,352)	standardized difference^b^	Sacubitril/valsartan (*n* = 3,276)	ACEI/ARB (*n* = 3,645)	standardized difference^b^
**Age at index date, years**	3,736	66.4 ± 15.6	64.9 ± 15.2	66.5 ± 15.6	-0.11	65.5 ± 14.6	66.5 ± 15.5	-0.07
**Age group (years)**	3,736							
20-49		582 (15.6)	62 (16.1)	520 (15.5)	0.02	367 (11.2)	559 (15.3)	-0.12
50-64		1,097 (29.4)	116 (30.2)	981 (29.3)	0.02	1,224 (37.4)	1,068 (29.3)	0.17
65-74		807 (21.6)	97 (25.3)	710 (21.2)	0.10	804 (24.5)	786 (21.6)	0.07
75-84		820 (21.9)	83 (21.6)	737 (22.0)	-0.01	626 (19.1)	808 (22.2)	-0.08
≥85		430 (11.5)	26 (6.8)	404 (12.1)	-0.18	256 (7.8)	425 (11.6)	-0.13
**Sex**	3,736							
Men		2,580 (69.1)	285 (74.2)	2,295 (68.5)	0.13	2,370 (72.3)	2,513 (68.9)	0.07
Women		1,156 (30.9)	99 (25.8)	1,057 (31.5)	-0.13	907 (27.7)	1,133 (31.1)	-0.07
**Smoking**	3,736	1,307 (35.0)	138 (35.9)	1,169 (34.9)	0.02	1,245 (38.0)	1,275 (35.0)	0.06
**Body mass index**	3,687	25.1 ± 5.2	25.5 ± 5.3	25.1 ± 5.2	0.09	24.5 ± 4.7	25.1 ± 5.2	-0.11
**Vital sign**								
Heart rate, beats/min	3,736	90.6 ± 21.8	88.3 ± 21.0	90.8 ± 21.8	-0.12	90.7 ± 23.5	90.6 ± 21.8	<0.01
Systolic blood pressure, mmHg	3,736	129.4 ± 24.2	128.1 ± 24.9	129.5 ± 24.1	-0.06	127.9 ± 20.6	129.4 ± 24.1	-0.06
Diastolic blood pressure, mmHg	3,736	79.7 ± 18.4	78.1 ± 17.8	79.9 ± 18.5	-0.10	79.2 ± 16.0	79.7 ± 18.5	-0.03
**Previous HF admissions in the previous year**	3,736	676 (18.1)	139 (36.2)	537 (16.0)	0.47	469 (14.3)	645 (17.7)	-0.09
**Number of HF admissions in the previous 3 years**	3,736							
0		2,671 (71.5)	201 (52.3)	2,470 (73.7)	-0.45	2,403 (73.4)	2,620 (71.9)	0.03
1		532 (14.2)	80 (20.8)	452 (13.5)	0.20	436 (13.3)	516 (14.2)	-0.02
2		266 (7.1)	48 (12.5)	218 (6.5)	0.21	241 (7.4)	257 (7.1)	0.01
≥ 3		267 (7.1)	55 (14.3)	212 (6.3)	0.27	195 (6.0)	252 (6.9)	-0.04
**Comorbidities**								
Hypertension		2,558 (68.5)	283 (73.7)	2,275 (67.9)	0.13	2,368 (72.3)	2,502 (68.6)	0.08
Diabetes		1,667 (44.6)	188 (49.0)	1,479 (44.1)	0.10	1,673 (51.1)	1,629 (44.7)	0.13
Dyslipidemia		1,857 (49.7)	205 (53.4)	1,652 (49.3)	0.08	1,586 (48.4)	1,811 (49.7)	-0.03
Atrial fibrillation		1,106 (29.6)	116 (30.2)	990 (29.5)	0.01	992 (30.3)	1,080 (29.6)	0.01
Myocardial infarction		608 (16.3)	78 (20.3)	530 (15.8)	0.12	766 (23.4)	598 (16.4)	0.18
Stroke		424 (11.3)	51 (13.3)	373 (11.1)	0.07	408 (12.4)	411 (11.3)	0.04
Coronary artery disease		1,210 (32.4)	113 (29.4)	1,097 (32.7)	-0.07	1,033 (31.5)	1,197 (32.8)	-0.03
Chronic obstructive pulmonary disease		548 (14.7)	84 (21.9)	464 (13.8)	0.21	507 (15.5)	536 (14.7)	0.02
**Baseline echocardiography**								
LVEF, %	3,477	29.4 ± 7.5	30.2 ± 11.4	29.3 ± 6.9	0.10	28.7 ± 9.1	29.3 ± 6.9	-0.08
LVEDD, mm	3,473	59.7 ± 8.6	61.9 ± 9.4	59.4 ± 8.5	0.28	60.1 ± 9.2	59.6 ± 8.5	0.05
LVESD, mm	3,472	50.5 ± 8.8	52.2 ± 10.6	50.3 ± 8.6	0.19	50.9 ± 9.8	50.4 ± 8.7	0.05
LA, mm	3,466	44.5 ± 8.0	45.3 ± 8.2	44.4 ± 8.0	0.10	44.4 ± 7.9	44.5 ± 8.0	<0.01
MR severity	3,736							
Trivial/None		367 (9.8)	32 (8.3)	335 (10.0)	-0.06	308 (9.4)	361 (9.9)	-0.02
Mild		1,798 (48.1)	178 (46.4)	1,620 (48.3)	-0.04	1,625 (49.6)	1,747 (47.9)	0.03
Moderate		946 (25.3)	120 (31.3)	826 (24.6)	0.15	968 (29.6)	926 (25.4)	0.09
Severe		334 (8.9)	45 (11.7)	289 (8.6)	0.10	324 (9.9)	325 (8.9)	0.03
Missing		291 (7.8)	9 (2.3)	282 (8.4)	-0.27	51 (1.5)	287 (7.9)	-0.30
**Baseline laboratory data**								
BNP, pg/mL	2,796	1340 [691, 2254]	1566 [795, 2781]	1320 [678, 2200]	0.19	1729 [766, 2807]	1333 [685, 2228]	0.19
BUN, mg/dL	3,579	29.8 ± 21.6	33.7 ± 25.2	29.3 ± 21.2	0.19	31.2 ± 21.0	29.6 ± 21.1	0.08
Serum creatinine, mg/dl c	3,399	1.6 ± 1.3	1.8 ± 1.4	1.6 ± 1.3	0.17	1.6 ± 1.0	1.6 ± 1.3	0.05
eGFR, mL/min/1.73m^2^^c^	3,399	58.0 ± 29.9	52.1 ± 27.0	58.7 ± 30.1	-0.23	57.1 ± 29.7	58.1 ± 29.8	-0.03
Renal function status	3,736							
≥60 ml/min		1,617 (43.3)	132 (34.4)	1,485 (44.3)	-0.20	1,302 (39.7)	1,579 (43.3)	-0.07
30–59 ml/min		1,315 (35.2)	144 (37.5)	1,171 (34.9)	0.05	1,064 (32.5)	1,292 (35.5)	-0.06
<30 ml/min		477 (12.8)	66 (17.2)	411 (12.3)	0.14	451 (13.8)	456 (12.5)	0.04
Dialysis		327 (8.8)	42 (10.9)	285 (8.5)	0.08	459 (14.0)	318 (8.7)	0.17
Sodium (Na), mEq/L	3,723	137.5 ± 4.6	136.9 ± 4.8	137.5 ± 4.6	-0.12	137.8 ± 4.3	137.5 ± 4.6	0.07
Potassium (K), mEq/L	3,725	4.0 ± 0.6	4.1 ± 0.7	4.0 ± 0.6	0.09	4.1 ± 0.7	4.0 ± 0.6	0.14
Hemoglobin, g/dL	3,729	12.7 ± 2.6	12.6 ± 2.5	12.7 ± 2.6	-0.07	12.8 ± 2.4	12.7 ± 2.6	0.02
**Hypoglycemic medications**								
Thiazolidinedione	3,736	40 (1.1)	3 (0.8)	37 (1.1)	-0.03	24 (0.7)	38 (1.1)	-0.03
GLP1RA	3,736	6 (0.2)	1 (0.3)	5 (0.1)	0.02	8 (0.23)	6 (0.16)	0.01
SGLT2i	3,736	206 (5.5)	36 (9.4)	170 (5.1)	0.17	196 (6.0)	199 (5.5)	0.02
**Other medications in the previous 3 months**								
Beta-blockers	3,736	3,255 (87.1)	333 (86.7)	2,922 (87.2)	-0.01	2,857 (87.2)	3,187 (87.4)	-0.01
MRAs	3,736	1,984 (53.1)	268 (69.8)	1,716 (51.2)	0.39	1,769 (54.0)	1,925 (52.8)	0.02
Ivabradine	3,736	605 (16.2)	118 (30.7)	487 (14.5)	0.39	551 (16.8)	576 (15.8)	0.03
Loop diuretics	3,736	3,151 (84.3)	335 (87.2)	2,816 (84.0)	0.09	2,835 (86.5)	3,082 (84.5)	0.06
Digoxin	3,736	780 (20.9)	97 (25.3)	683 (20.4)	0.12	526 (16.1)	760 (20.9)	-0.12
Amiodarone	3,736	570 (15.3)	83 (21.6)	487 (14.5)	0.18	656 (20.0)	554 (15.2)	0.13
**Other treatments**								
Implantable cardioverter-defibrillator	3,736	120 (3.2)	22 (5.7)	98 (2.9)	0.14	147 (4.5)	118 (3.2)	0.06
CRT	3,736	40 (1.1)	11 (2.9)	29 (0.9)	0.15	26 (0.79)	37 (1.00)	-0.02
**In-hospital event**								
Hospital days	3,736	13.0 ± 14.7	17.4 ± 20.1	12.5 ± 13.9	0.28	14.2 ± 13.1	12.9 ± 15.9	0.09
ICU days	3,736	1.9 ± 5.2	3.1 ± 8.5	1.7 ± 4.7	0.19	1.9 ± 5.6	1.8 ± 5.1	<0.01
Inotropes	3,736	583 (15.6)	82 (21.4)	501 (14.9)	0.17	663 (20.3)	564 (15.5)	0.13
Intubation	3,736	97 (2.6)	21 (5.5)	76 (2.3)	0.17	111 (3.4)	94 (2.6)	0.05
Acute myocardial infarction	3,736	486 (13.0)	38 (9.9)	448 (13.4)	-0.11	399 (12.2)	483 (13.3)	-0.03
PCI	3,736	581 (15.6)	54 (14.1)	527 (15.7)	-0.05	639 (19.5)	573 (15.7)	0.10

Abbreviations: ACEI/ARB, angiotensin-converting enzyme inhibitor/angiotensin receptor blocker; HF, heart failure; LVEF, left ventricular ejection fraction; LVEDD, left ventricular end-diastolic diameter; LVESD, left ventricular end-systole diameter; LA, left atrial; MR, mitral regurgitation; BNP, B-type natriuretic peptide; BUN, blood urine nitrogen; eGFR, estimated glomerular filtration rate; GLP1RA, glucagon-like peptide-1 receptor agonist; SGLT2i, sodium glucose cotransporter 2 inhibitor; MRA, mineralocorticoid antagonist; CRT, cardiac resynchronization therapy; ICU, intensive care unit; PCI, percutaneous coronary intervention.

a All covariates listed were used to calculate the propensity score. Values are presented as n (%).

b An absolute standardized difference of < 0.1 indicated a negligible difference, and a value between 0.1 and 0.2 is considered as a small difference.

c Patients with dialysis at baseline were excluded.

We compared the risks of fatal time-to-event outcomes (i.e., all-cause death and the composite outcome of all-cause death and rehospitalization for HF) between the groups using a Cox proportional hazard model. For non-fatal outcomes (i.e., rehospitalization for HF, non-fatal MI, non-fatal stroke and safety outcomes), the risk between groups was compared using a Fine and Gray subdistribution hazard model that considered death as a competing risk. To further rule out possible residual confounding even after IPTW, we further adjusted for the covariates with an absolute STD value >0.1 in the aforementioned survival models, including age, BMI, diabetes, prior MI, mitral regurgitation severity, BNP, dialysis, potassium, digoxin, amiodarone, inotropic agents and PCI during the index admission. To avoid prevalent user bias in the ACEI/ARB group (prevalent ACEI/ARB users were not excluded in the primary analysis), we adopted a new user design for both the sacubitril/valsartan and ACEI/ARB groups as a sensitivity analysis [12]. IPTW based on propensity score was conducted again on this new cohort. We also performed another sensitivity analysis using multivariable adjustments in the original cohort with imputation. The three main effectiveness outcomes at the end of follow-up were assessed in the sensitivity analyses, including all-cause death, rehospitalization for HF and the composite outcome of both.

Subgroup analysis was performed to determine whether the HRs of the primary composite outcome for the sacubitril/valsartan and ACEI/ARB groups were consistent among the prespecified subgroups, which included age (<75 years or ≥75 years), sex, smoking, BMI (<27 or ≥27 kg/m^2^), systolic blood pressure (≤129 or >129 mmHg), previous HF admission in the previous 3 years, hypertension, diabetes, atrial fibrillation, chronic obstructive pulmonary disease, LVEF (≤30% or >30%), mitral regurgitation severity (i.e., none/mild vs. moderate/severe), BNP (≤1340 or >1340 pg/mL; by median value), renal function (i.e., ≥60 mL/min/1.73 m^2^, 30–59 mL/min/1.73 m^2^, <30 mL/min/1.73 m^2^, or dialysis), the use of beta-blockers, ivabradine, or sodium-glucose cotransporter 2 inhibitors, and PCI during the index admission. The levels of NT-pro BNP at baseline and after 12 months of follow-up were compared using the Wilcoxon signed-rank test for each study group in the IPTW cohort. The change of NT-pro BNP level from baseline to 12^th^ month between groups was compared using generalized estimating equation (GEE) which included intercept, main effects of treatment group and time point and an interaction term of group by time. Another subgroup analysis was performed to determine whether the subdistribution hazard ratios (SHRs) of worsening renal function and progression to ESRD for the sacubitril/valsartan and ACEI/ARB groups were similar in the prespecified renal function subgroups (i.e., ≥60 mL/min/1.73 m^2^ or <60 mL/min/1.73 m^2^; excluding patients with dialysis at baseline). We also compared the risk of the primary composite outcome in the sacubitril/valsartan users among those who could tolerate doses of ≥200 mg/day, 100 mg, or ≤50 mg at baseline and 3 months using a Cox model in the original cohort without adjusting for covariates (due to the relatively low number of patients). Finally, we compared the cumulative incidence of the composite outcome between the ACEI and ARB groups using a Cox proportional hazard model with traditional multivariable adjustments (the adjusted covariates are listed in Table 1) in the original cohort. All statistical analyses were performed using SAS Version 9.4 (SAS Institute, Cary, NC, USA). All statistical tests were 2-sided, and a *P* value < 0.05 was considered to be significant. Data were analyzed from January 2020 through March 2021.

### 2.6 Role of the funding source

The funders had no role in the design and conduct of the study; collection, management, analysis, and interpretation of the data; preparation, review, or approval of the manuscript; and decision to submit the manuscript for publication.

## 3 Results

### 3.1 Patient characteristics and baseline demographics

The mean follow-up period was 11.8 months (SD, 12.3 months), and the maximum follow-up duration was 3.6 years. The patients in the sacubitril/valsartan group had higher rates of previous HF admissions in the previous year, hypertension, prior MI, chronic obstructive pulmonary disease, cardioverter–defibrillator implantation, cardiac resynchronization therapy, use of inotropic agents and intubation, and longer index HF admission days and ICU stay during the index admission than the patients in the ACEI/ARB group. The patients in the sacubitril/valsartan group also had larger LV end-diastolic diameter and LV end-systolic diameter, higher BNP level, higher serum creatinine level, and lower eGFR than the patients in the ACEI/ARB group. After IPTW, the demographics, comorbidities, and medications at baseline were well balanced except for some variables (e.g., age, BMI, diabetes, prior MI, BNP, dialysis, potassium, digoxin, amiodarone, inotropic agents, and PCI) with absolute STD values larger than 0.1 but less than 0.2 (Table 1).

### 3.2 Effectiveness Outcomes

**Fig. 2:**
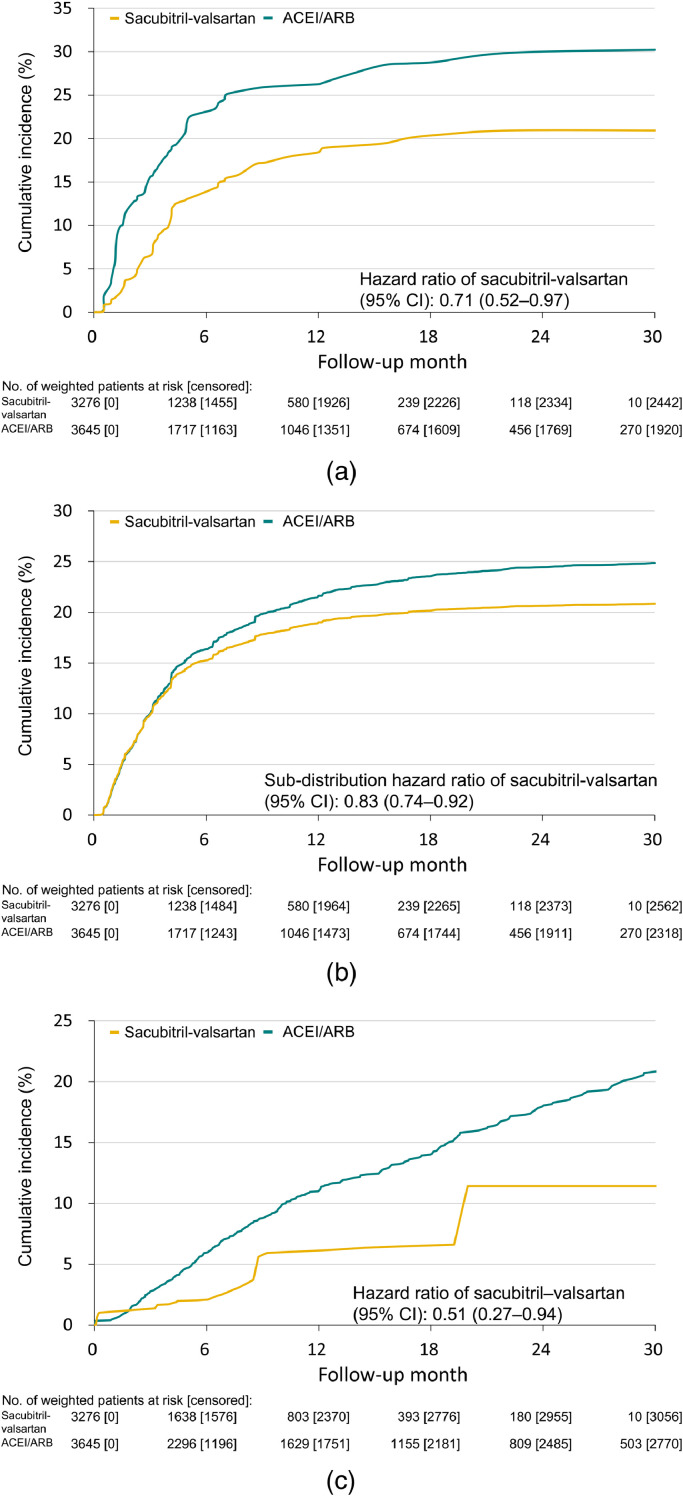
Cumulative incidence rates of the outcomes. (a) Composite of all-cause death and heart failure rehospitalization, (b) heart failure rehospitalization, and (c) all-cause death in the IPTW cohort. The survival time was truncated at the 30^th^ month in the plot due to the small number of remaining patients at risk. Cumulative event rate is presented as the composite outcome and all-cause death. Cumulative incidence function in a Fine and Gray subdistribution hazard model is presented for heart failure rehospitalization. ACEI/ARB, angiotensin-converting enzyme inhibitor/angiotensin receptor blocker; CI, confidence interval; HR, hazard ratio; IPTW, inverse probability of treatment weighting.

The effectiveness outcomes are shown in Table 2, and the cumulative incidence rates of the outcomes are shown in Fig. 2. The composite of rehospitalization for HF and death occurred in 22.9% of the patients in the sacubitril/valsartan group and 32.6% of the patients in the ACEI/ARB group. The absolute endpoint reduction was 9.7% (HR 0.71, 95% CI 0.52–0.97). The sacubitril/valsartan group had significantly lower risks of rehospitalization for HF (SHR 0.83, 95% CI 0.74–0.92) and all-cause death (HR 0.51, 95% CI 0.27–0.94). A greater reduction in the risk of rehospitalization for HF in the sacubitril/valsartan group compared to the ACEI/ARB group was evident as early as 6 months (SHR 0.83, 95% CI, 0.74–0.92), and the benefit of risk reduction persisted until the end of the study. Similarly, we observed a significantly lower mortality rate in the sacubitril/valsartan group after 6 months of follow-up (HR 0.34, 95% CI 0.13–0.90), which remained significant throughout the study. The risks of non-fatal MI (SHR 0.86, 95% CI 0.62–1.18) and non-fatal ischemic stroke did not differ significantly between the two study groups (SHR 0.50, 95% CI 0.24–1.04).

**Table 2:** Effectiveness and safety clinical outcomes.

Outcome	Data after IPTW^a^			
	Sacubitril/valsartan	ACEI/ARB	HR or SHR for Sacubitril/valsartan (95% CI)^b^	*P* value
**Effectiveness outcomes at 6 months**				
Composite of rehospitalization for HF and death	583 (17.8)	765 (21.0)	0.78 (0.55–1.12)	0.18
Death	62 (1.9)	153 (4.2)	0.34 (0.13–0.90)	0.02
Rehospitalization for HF	554 (16.9)	685 (18.8)	0.83 (0.74–0.92)	0.001
Non-fatal myocardial infarction	69 (2.1)	73 (2.0)	0.86 (0.62–1.18)	0.34
Non-fatal ischemic stroke	10 (0.30)	28 (0.77)	0.50 (0.24–1.04)	0.06
**Effectiveness outcomes at the end of the study**				
Composite of rehospitalization for HF and death	750 (22.9)	1188 (32.6)	0.71 (0.52–0.97)	0.03
Death	210 (6.4)	372 (10.2)	0.51 (0.27–0.94)	0.03
Rehospitalization for HF	704 (21.5)	1057 (29.0)	0.83 (0.74–0.92)	0.001
Non-fatal myocardial infarction	82 (2.5)	120 (3.3)	0.86 (0.62–1.18)	0.34
Non-fatal ischemic stroke	11 (0.34)	69 (1.9)	0.50 (0.24–1.04)	0.06
**Safety outcomes**^c^				
Worsening renal function d	744 (22.7)	878 (24.1)	1.08 (0.97–1.20)	0.16
Composite of decline of eGFR >50% or progression to ESRD	498 (15.2)	718 (19.7)	0.97 (0.84–1.13)	0.73
Decline in eGFR >50% from baseline	288 (8.8)	477 (13.1)	0.93 (0.79–1.09)	0.35
Progression to ESRD	278 (8.5)	328 (9.0)	1.15 (0.86–1.53)	0.33
Creatinine ≥2.5 mg/dL	793 (24.2)	755 (20.7)	0.94 (0.81–1.08)	0.35
Creatinine ≥3 mg/dL	678 (20.7)	612 (16.8)	1.12 (0.95–1.31)	0.18
Potassium ≥6 mg/dL	174 (5.3)	230 (6.3)	1.07 (0.84–1.36)	0.59

Abbreviations: ACEI/ARB, angiotensin-converting enzyme inhibitor/angiotensin receptor blocker; CI, confidence interval; eGFR, estimated glomerular filtration rate; ESRD, end-stage renal disease, HF, heart failure; HR, hazard ratio; IPTW, inverse probability of treatment weighting; SHR, subdistribution hazard ratio.

a Values are presented as n (%).

b Additionally adjusted for age, body mass index, diabetes, myocardial infarction, mitral regurgitation severity, B-type natriuretic peptide, renal function status, potassium, digoxin, amiodarone, inotropic agents and percutaneous coronary intervention.

c Patients with dialysis at baseline were excluded.

d An increase in creatinine more than 0.5 and a decrease in eGFR more than 25%.

### 3.3 Sensitivity analysis of effectiveness outcomes

After excluding 1429 prevalence users of ACEIs or ARBs in the ACEI/ARB group, there were 1923 new users who initiated ACEI/ARB therapy during the index HF admission (eTable 2). The results of new user analysis showed that the sacubitril/valsartan group had significantly lower risks of death (HR 0.52, 95% CI 0.27–0.99) and rehospitalization for HF (SHR 0.84, 95% CI 0.74–0.94) (eTable 3). The sacubitril/valsartan group also had a lower risk of the composite outcome, although the difference was not statistically significant, possibly due to the smaller sample size compared to the primary analysis. The results using multivariable adjustments on the original cohort showed a benefit in the sacubitril-valsartan group but without statistical significance, possibly due to over-fitting as more than 40 covariates were adjusted for in the analysis (eTable 4).

### 3.4 Safety outcomes

The risks of worsening renal function, a decline in eGFR ≥50% from baseline, progression to ESRD, elevation of creatinine of ≥2.5 mg/dL or ≥3 mg/dL, and severe hyperkalemia did not differ significantly between the sacubitril/valsartan group and ACEI/ARB group (Table 2). The risks of worsening renal function and progression to ESRD were similar between the subgroups with chronic kidney disease (CKD) (eGFR <60 mL/min/1.73 m^2^) and without CKD (eGFR ≥60 mL/min/1.73 m^2^) (*P* for interaction = 0.53 and 0.49, respectively) (eFig. 1 in the Supplement).

### 3.5 Subgroup analysis

**Fig. 3:**
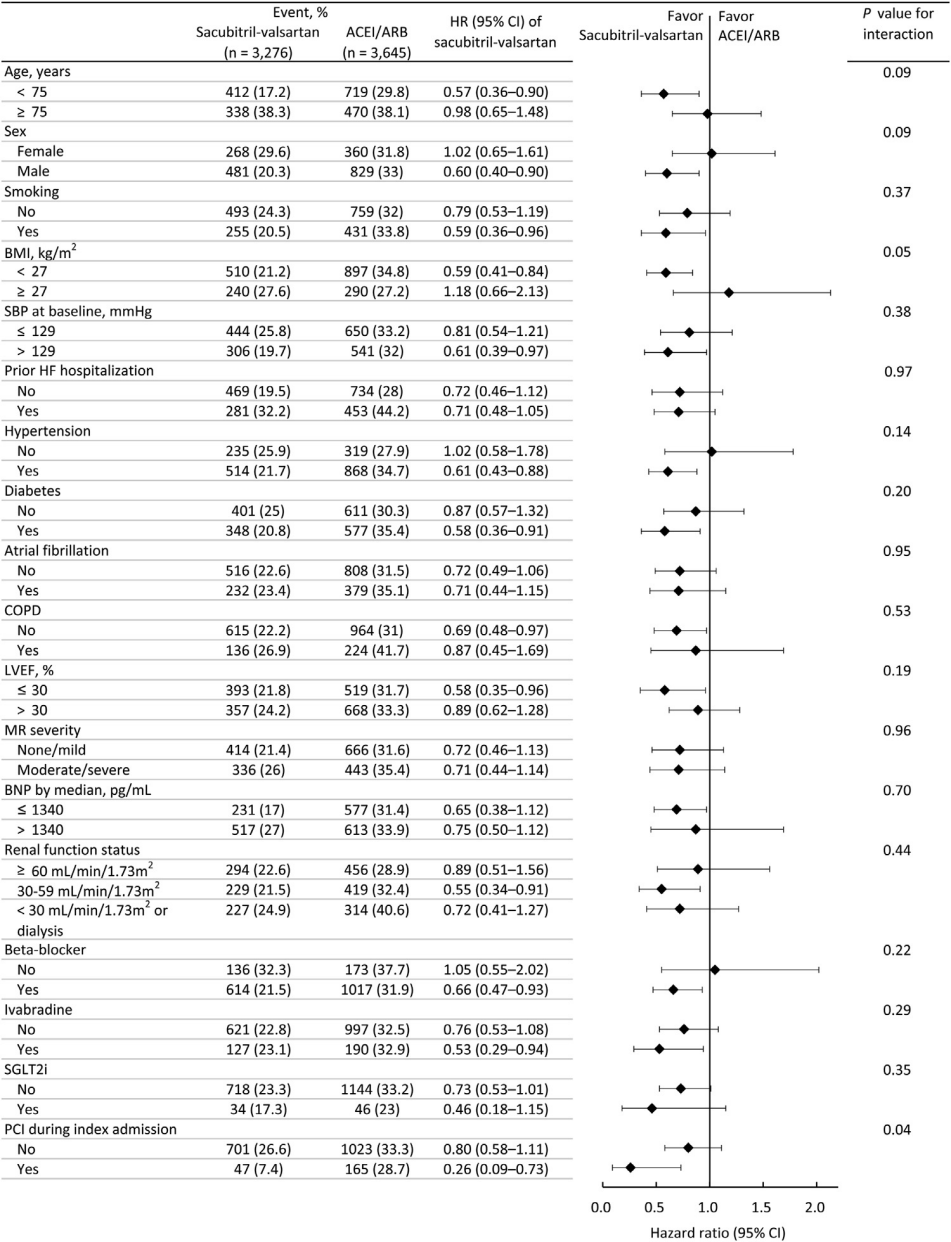
Composite outcomes of rehospitalization for heart failure and all-cause death by subgroups in the IPTW cohort. IPTW, inverse probability of treatment weighting; HR, hazard ratio; BMI, body mass index; LVEF, left ventricular ejection fraction; BNP, B type natriuretic peptide; MRA, mineralocorticoid receptor antagonist; SGLT2i, sodium-glucose cotransporter 2 inhibitor; AMI, acute myocardial infarction; PCI, percutaneous coronary intervention; ICU, intensive care unit. Of note, the significance levels of the subgroup analyses were “1” except for BMI (*P* for interaction = 0.83) and PCI (*P* for interaction = 0.72) after Bonferroni correction for type I error inflation.

In the subgroup analysis, we observed that the risk of the composite of rehospitalization for HF and death remained constant across all planned subgroups except for BMI and PCI during the index admission (Fig. 3). The observed beneficial effect of sacubitril/valsartan was more apparent in those with a BMI <27 kg/m^2^ and those who received a PCI during the index admission (*P* for interaction = 0.05 and 0.04, respectively). However, it should be noted that the results were insignificant after Bonferroni correction for type I error inflation.

### 3.6 Additional analysis

**Fig. 4:**
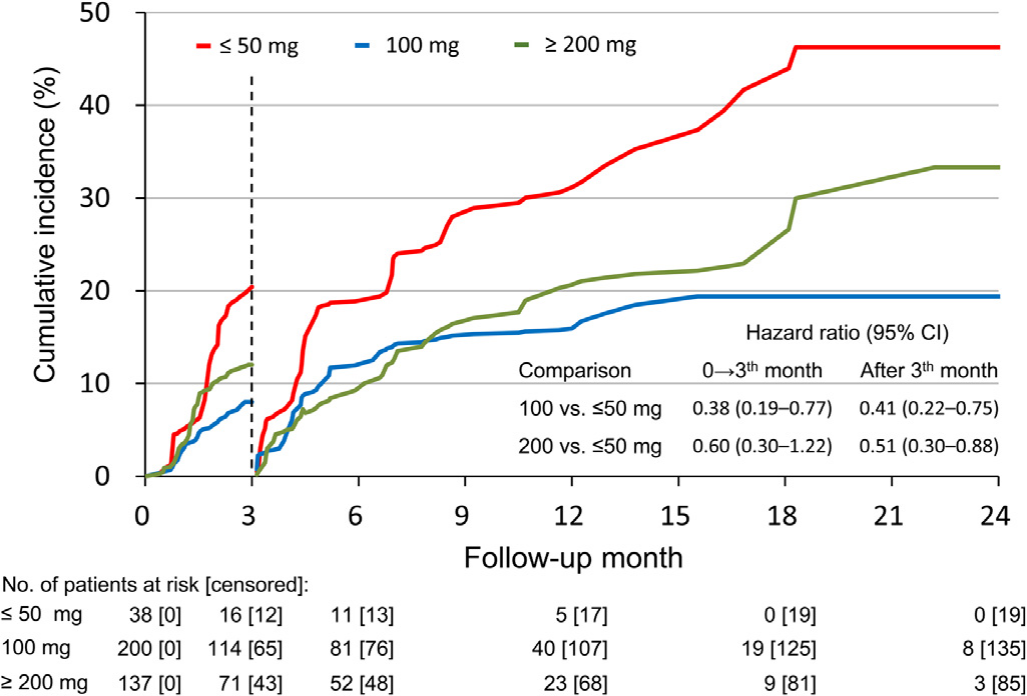
Cumulative incidence of rehospitalization for heart failure and death by sacubitril/valsartan dose in the original cohort. ACEI/ARB, angiotensin-converting enzyme inhibitor/angiotensin receptor blocker

We examined changes in NT-pro BNP level at baseline and after 12 months of follow-up in 42 patients in the sacubitril/valsartan group and 133 patients in the ACEI/ARB group. Although the NT-pro BNP level significantly decreased in both study groups at 12 months of follow-up (*P* < 0.001 in both groups), there was a significantly greater reduction in the sacubitril/valsartan group (3571 to 1707 pg/mL in the sacubitril/valsartan group vs. 2282 to 1631 pg/mL in the ACEI/ARB group, *P* for interaction in GEE model = 0.001) (eFig. 2 in the Supplement). The daily prescribed doses of sacubitril/valsartan at baseline, 3 months, 12 months, and the end of the study are provided in eTable 5 in the Supplement. Except for 9 patients who had missing dose data at baseline, the drug cessation rates were 26.1%, 28%, and 28% at 3 months, 12 months, and the end of the study, respectively. The mean daily doses of sacubitril/valsartan at baseline and 3 months of follow-up were 145.7 and 160.5 mg, respectively. The unadjusted cumulative event rates of the composite of rehospitalization for HF and death stratified by the ability to tolerate doses of ≥200 mg/day, 100 mg/day, or ≤50 mg/day at baseline and 3 months are shown in Fig. 4. The patients who could tolerate a dose of 100 mg/day or ≥200 mg/day at 3 months had lower composite outcomes than those who received a dose of ≤50 mg/day (HR 0.41, 95% CI 0.22–0.75; and HR 0.51, 95% CI 0.30–0.88, respectively). Finally, no significant differences were found in the cumulative incidence rates of rehospitalization for HF or death between the ACEI and ARB groups (eFig. 3 in the Supplement).

## 4 Discussion

In this real-world cohort study, we evaluated the effectiveness and safety associated with initiating sacubitril/valsartan treatment compared to ACEI/ARB treatment in patients with HFrEF during the high-risk transition period following acute HF hospitalization. We found that the patients who initiated sacubitril/valsartan therapy had a significantly lower risk of the primary composite outcome of rehospitalization for HF and death than the patients who received ACEI/ARB therapy. The decrease in rehospitalization for HF and death associated with the initiation of sacubitril/valsartan was clinically relevant, as there was a significantly lower risk at 6 months of follow-up that persisted until the end of the study. Before IPTW, the patients in the sacubitril/valsartan group were sicker with a larger LV end-diastolic diameter and LV end-systolic diameter, higher BNP level, higher serum creatinine level, and lower eGFR than the patients in the ACEI/ARB group. However, we did detect a lower risk of rehospitalization for HF and death in the sacubitril/valsartan group than the ACEI/ARB group. The safety outcome of worsening renal function, decline in eGFR >50% from baseline, progression to ESRD, and severe hyperkalemia were similar between the two study groups. These findings may help to guide clinicians with regards to the optimal therapy for patients with acute HF after hemodynamic stabilization.

The lower rates of rehospitalization for HF and death associated with sacubitril/valsartan therapy in this study are consistent with the PIONEER-HF trial [4]. However, the PIONEER-HF trial excluded patients with baseline eGFR levels of <30 mL/min/1.73 m^2^, whereas 12.8% of our study population had an eGFR <30 mL/min/1.73 m^2^ and 8.8% were receiving dialysis. Approximately one-third of our patients had moderate or severe mitral regurgitation, 13% had acute MI during the index hospitalization, and 15.6% received a PCI. Patients with these characteristics were also excluded from the PIONEER-HF trial [3]. Therefore, our study included a wider range of acute HF patients, which is more applicable to real-world clinical practice. Furthermore, the majority of our reference group (62.2%, 2085/3352) received ARBs, whereas the only reference group in the PIONEER-HF trial received ACEIs. Although ACEIs and ARBs have been incorporated into guidelines for the treatment of HF by international cardiology societies, the prescription rate of ARBs compared to ACEIs is relatively high in Taiwan [13]. The prescription ratio of ACEIs to ARBs in the current study is consistent with the Taiwan Society of Cardiology (TSOC) HFrEF registry, which showed a higher prescription rate of ARBs compared to ACEIs (34.6% vs 27.5%) in patients with HFrEF [14]. A possible reason for this finding may be related to the high prevalence of ACEI-associated cough in Chinese populations [5]. Nonetheless, because our population had a higher prescription rate of ARBs, we were able to evaluate and understand the clinical outcomes of sacubitril/valsartan vs ARB treatment in patients with acute HF. There were no significant differences in the cumulative incidence rates of rehospitalization for HF or death among the different types of ACEIs or ARBs. Taken together, this study adds to the knowledge regarding evidence with longer follow-up duration for the in-hospital initiation of sacubitril/valsartan compared with ACEI/ARB treatment in patients who are hospitalized for acute HF.

There was a significantly greater reduction in NT-pro BNP level in the sacubitril/valsartan group than in the ACEI/ARB group at 12 months of follow-up. This is consistent with previous analyses of data from the PARADIGM-HF trial and PIONEER-HF trial [3,15]. NT-proBNP is a biomarker of hemodynamic stress and neurohormonal activation, and it has prognostic value in patients with HF [16]. The recent PROVE-HF study demonstrated that the reduction in NT-proBNP following sacubitril/valsartan treatment was associated with reductions in left atrial volume index, and ratio of early transmitral Doppler velocity/early diastolic annular velocity (E/e’), which reflect improvements in elevated cardiac filling pressures and are important prognostic factors in patients with HF [17]. Consistent with these reports, the greater reduction in NT-pro BNP in the sacubitril/valsartan group in the present study may reflect that the patients had favorable cardiac improvements and consequently lower rates of rehospitalization for HF and death.

Another important finding of this study is the safety profile of sacubitril/valsartan in the context of acute HF in longer follow-up duration. Of note, the mean serum creatinine level of the study population was 1.6 mg/dl, and more than half had an eGFR <60 mL/min/1.73 m^2^. However, the rates of worsening renal function, a decline in eGFR >50% from baseline, progression to ESRD, and severe hyperkalemia did not differ significantly between the sacubitril–valsartan and ACEI/ARB groups. Furthermore, the risks of worsening renal function and progression to ESRD were similar between the subgroups with or without CKD (eGFR ≥60 mL/min/1.73 m^2^) (*P* for interaction = 0.52 and 0.49, respectively). This provides evidence of the renal safety of initiating sacubitril/valsartan in patients with acute HF and renal dysfunction, who are at high risk of worsening renal function.

In this study, almost one-sixth of the patients received a PCI during the index hospitalization, however such patients were excluded from the PIONEER-HF trial. Interestingly, we found that the beneficial effect of sacubitril/valsartan treatment on rehospitalization for HF and death was more apparent in the patients who received a PCI during the index admission than in those who did not. This is consistent with a report by Torrado et al. who investigated sacubitril–valsartan in a model of ischemia-reperfusion in rabbits to mimic the clinical events in acute MI patients receiving a coronary intervention [18,19]. They found superior short-term and long-term benefits in preventing MI-induced LV dysfunction with sacubitril/valsartan compared to valsartan. Another study by Zhang et al. also demonstrated a lower readmission rate, smaller infarction size, and higher LVEF with sacubitril/valsartan treatment compared with ACEIs at 6 months in patients with ST-elevation MI after primary PCI [20]. The exact mechanism associated with the beneficial effect of sacubitril/valsartan in patients receiving PCI is unclear, however several potential mechanisms have been postulated. First, sacubitril/valsartan treatment may involve an early reduction in LV wall stress through its hemodynamic effect on reducing afterload [21]. Second, acute HF is associated with elevated cardiac filling pressures, which could decrease the gradient of blood flow to the subendocardial tissue during diastole. If coronary artery stenosis is present, the elevation of LV end-diastolic filling pressure (LVEDP) may seriously jeopardize the vulnerable subendocardium. Sacubitril/valsartan treatment has been associated with a reduction in indexed LV and left atrium volumes and E/e’, which are parameters of LVEDP [17,22]. A reduction in LVEDP may affect the improvement in coronary perfusion. Finally, inhibition of the breakdown of C-type natriuretic peptide locally and increased intracellular cyclic GMP concentration of sacubitril/valsartan may involve the regulation of coronary blood flow and ameliorate myocardial damage [18,21]. Further prospective studies are warranted to investigate this issue.

In our analysis, most of the patients (63.4%, 238/375) did not receive the recommended standard dose (<200 mg/day) of sacubitril/valsartan after stabilization for acute HF, and less than half (45.8%, 127/277) reached the recommended standard dose (≥200 mg/day) at 3 months. The initial underdose of sacubitril/valsartan at baseline is consistent with the PIONEER-HF trial and may reflect the severe clinical profile of patients recently admitted for acute HF [23]. At 3 months, compared to the patients who received sacubitril/valsartan ≤50 mg/day, those who could tolerate a dose of 100 mg/day or ≥200 mg/day had a lower risk of the composite outcome through to the end of study. There was no significant difference in the risk of composite outcome between those receiving 100 mg/day or ≥200 mg/day. This is consistent with the dose analysis in the PIONEER-HF trial, in which the efficacy and safety of sacubitril/valsartan was generally consistent across doses of 100 mg/day, 200 mg/day, or 400 mg/day [23]. These data support that a lower dose (100 mg/day) of sacubitril/valsartan may also be clinically beneficial during the vulnerable post-hospitalization period in patients with HFrEF.

This study has several limitations. First, because of the retrospective nature of the study, the two study groups may have had inherent differences. To reduce selection bias, we used propensity score weighting to balance differences associated with major characteristics at baseline. To further rule out possible residual confounding even after IPTW, we further adjusted for the covariates with absolute STD values >0.1 in the survival models. However, we still could not exclude the possibility of residual confounding or unmeasured confounding factors. Second, we did not have regular echocardiography follow-up data, which may have provided more direct evidence of cardiac improvement in the patients treated with sacubitril–valsartan. In addition, we did not have concomitant pharmacological treatment data during the follow-up period, which could have affected the clinical outcomes. Third, because of the relatively low number of patients, we could not adjust for covariates when comparing the risk of the primary composite outcome in the sacubitril/valsartan users among those who could tolerate doses of ≥200 mg/day, 100 mg, or ≤50 mg at baseline and 3 months. Fourth, not all of the patients had NP-pro BNP data at baseline and 12 months of follow-up. Finally, underestimation resulting from noncompliance is likely, because information on prescribed drugs may not reflect the actual use.

In conclusion, compared with ACEI/ARB therapy, initiating sacubitril–valsartan therapy in patients who were hospitalized for acute HF was associated with lower rates of rehospitalization for HF and death, with no increase in worsening renal function or severe hyperkalemia events. These results support the initiation of sacubitril/valsartan among patients with HFrEF who are hospitalized for acute HF and as an alternative to ACEIs/ARBs in real-world practice. However, it should be noted that the sensitivity analysis by multivariable covariates adjustment revealed less significant results. Therefore, further prospective studies are needed to confirm these findings.

## 5 Contributions

Dr. I-Chang Hsieh and Dr. Ming-Jer Hsieh had full access to all study data and takes responsibility for the integrity of the data and the accuracy of the data analysis. Dr. I-Chang Hsieh and Dr. Ming-Jer Hsieh contributed equally as corresponding authors.

Concept and design: Dong-Yi Chen, I-Chang Hsieh, Ming-Jer Hsieh

Acquisition, analysis, or interpretation of data: Dong-Yi Chen, Ming-Jer Hsieh, I-Chang Hsieh

Drafting of the manuscript: Dong-Yi Chen, Ming-Jer Hsieh

Critical revision of the manuscript for important intellectual content: Dong-Yi Chen, Ming-Jer Hsieh, I-Chang Hsieh

Statistical analysis: Dong-Yi Chen, Shao-Wei Chen, Wen-Kuan Huang

Obtained funding: Dong-Yi Chen, I-Chang Hsieh

Administrative, technical, or material support: Shang-Hung Chang, Chi-Nan Tseng, Wen-Kuan Huang

Supervision: Chun-Chi Chen, I-Chang Hsieh, Ming-Shien Wen

## Declaration of Competing Interest

The authors declare that there is no conflict of interest.

## Appendix Supplementary materials

Image, application 1

Image, application 2
