# Supplementary material for: Clinical outcomes of Sacubitril/Valsartan in patients with acute heart failure: A multi-institution study
Source: eClinicalMedicine. 2021 Oct 8;41:101149. doi: 10.1016/j.eclinm.2021.101149 (PMC8515399; doi:10.1016/j.eclinm.2021.101149)
Supplement: Supplementary file 1 [file mmc1.docx]

**Caption for Supplementary Material**

**eTable 1. Codes used to define heart failure and outcomes in the study cohort**

**eTable 2. Baseline demographics and clinical characteristics of the study patients in the new user analysis**

**eTable 3. Effectiveness clinical outcomes at the end of follow-up in the new user analysis**

**eTable 4. Effectiveness and safety clinical outcomes in multivariable adjustment analysis**

**eTable 5. The daily prescribed dose of sacubitril–valsartan at baseline, 3 months, 12 months, and end of follow-up.**

**eFigure 1. Safety outcomes by renal function subgroups in the IPTW cohort.**

**eFigure 2. Level of NT-pro BNP at baseline and 12 months of follow-up in the IPTW cohort.**

**eFigure 3. Cumulative incidence of rehospitalization for HF and death by ACEI or ARB subtype in the original Cohort.**
