# Supplementary material for: Clinical outcomes of Sacubitril/Valsartan in patients with acute heart failure: A multi-institution study
Source: eClinicalMedicine. 2021 Oct 8;41:101149. doi: 10.1016/j.eclinm.2021.101149 (PMC8515399; doi:10.1016/j.eclinm.2021.101149)

**eTable 1. Codes used to define heart failure and outcomes in the study cohort**

| **Diagnosis** | **International Classification of Diseases 9th revision (ICD-9) code** | **International Classification of Diseases 10th revision (ICD-10) code** |
| --- | --- | --- |
| Heart failure | 428.xx | I50 |
| Hypertension | 401.xx-405.xx | I10-I15, N262 |
| Diabetes | 250.xx | E08-E13 |
| Dyslipidemia |  |  |
| Atrial fibrillation | 427.3x | I48 |
| Myocardial infarction | 410.xx, 412.xx | I21-I22 |
| Acute myocardial infarction | 410.xx | I21 |
| Stroke | 430.xx-437.xx | I60-I62, I66, I65.1, I65.0, I65.8, I65.9, I63.6, I63.8, I63.9, G45.0, G45.8, G45.1, G45.2, G46.0, G46.1, G46.2, G45.9, G45.4, G46.3, G46.4, G46.5, G46.6, G46.7, G46.8, I67.0, I67.1, I67.2, I67.4, I67.5, I67.6, I67.7, I67.9, I68.0, I68.2, I68.8 |
| Coronary artery disease | 410.xx-414.xx | I20-I24 |
| Chronic obstructive pulmonary disease | 491.xx, 492.xx, 496.xx | J41-J44 |
| Ischemic stroke | 433.xx–437.xx | I66, I65.1, I65.0, I65.8, I65.9, I63.6, I63.8, I63.9, G45.0, G45.8, G45.1, G45.2, G46.0, G46.1, G46.2, G45.9, G45.4, G46.3, G46.4, G46.5, G46.6, G46.7, G46.8, I67.0, I67.1, I67.2, I67.4, I67.5, I67.6, I67.7, I67.9, I68.0, I68.2, I68.8 |

**eTable 2. Baseline demographics and clinical characteristics of the study patients in the new user analysis**

|  | **Valid n** | **Before propensity score weighting^a^** | | | **After propensity score weighting^a^** | | |
| --- | --- | --- | --- | --- | --- | --- | --- |
| **Variable** |  | **Sacubitril/valsartan**  **(n = 384)** | **ACEI/ARB**  **(n = 1,923)** | **standardized difference^b^** | **Sacubitril/valsartan**  **(n = 2,015)** | **ACEI/ARB**  **(n = 2,218)** | **standardized difference^b^** |
| **Age at index date, years** | 2,307 | 64.9 ± 15.2 | 64.9 ± 16.3 | <0.01 | 64.2 ± 15.6 | 65.1 ± 16.0 | -0.06 |
| **Age group (years)** | 2,307 |  |  |  |  |  |  |
| 20-49 |  | 62 (16.1) | 358 (18.6) | -0.07 | 309 (15.3) | 393 (17.7) | -0.06 |
| 50-64 |  | 116 (30.2) | 600 (31.2) | -0.02 | 725 (36.0) | 696 (31.4) | 0.10 |
| 65-74 |  | 97 (25.3) | 358 (18.6) | 0.16 | 465 (23.1) | 429 (19.3) | 0.09 |
| 75-84 |  | 83 (21.6) | 388 (20.2) | 0.04 | 340 (16.9) | 458 (20.7) | -0.10 |
| ≥85 |  | 26 (6.8) | 219 (11.4) | -0.16 | 177 (8.8) | 242 (10.9) | -0.07 |
| **Sex** | 2,307 |  |  |  |  |  |  |
| Men |  | 285 (74.2) | 1,309 (68.1) | 0.14 | 1,449 (71.9) | 1,532 (69.1) | 0.06 |
| Women |  | 99 (25.8) | 614 (31.9) | -0.14 | 566 (28.1) | 686 (30.9) | -0.06 |
| **Smoking** | 2,307 | 138 (35.9) | 651 (33.9) | 0.04 | 730 (36.2) | 753 (33.9) | 0.05 |
| **Body mass index** | 2,265 | 25.5 ± 5.3 | 25.4 ± 5.6 | 0.02 | 24.8 ± 5.1 | 25.4 ± 5.5 | -0.11 |
| **Vital sign** |  |  |  |  |  |  |  |
| Heart rate, beats/min | 2,307 | 88.3 ± 21.0 | 94.8 ± 22.0 | -0.31 | 94.9 ± 24.3 | 94.0 ± 22.0 | 0.04 |
| Systolic blood pressure, mmHg | 2,307 | 128.1 ± 24.9 | 130.8 ± 24.6 | -0.11 | 128.6 ± 21.3 | 130.3 ± 24.4 | -0.08 |
| Diastolic blood pressure, mmHg | 2,307 | 78.1 ± 17.8 | 82.5 ± 19.4 | -0.23 | 80.2 ± 16.1 | 82.0 ± 19.2 | -0.10 |
| **Previous HF admissions in the previous year** | 2,307 | 139 (36.2) | 159 (8.3) | 0.71 | 266 (13.2) | 269 (12.1) | 0.03 |
| **Number of HF admissions in the previous 3 years** | 2,307 |  |  |  |  |  |  |
| 0 |  | 201 (52.3) | 1,634 (85.0) | -0.75 | 1,540 (76.4) | 1,780 (80.2) | -0.09 |
| 1 |  | 80 (20.8) | 172 (8.9) | 0.34 | 268 (13.3) | 241 (10.9) | 0.08 |
| 2 |  | 48 (12.5) | 67 (3.5) | 0.34 | 106 (5.3) | 110 (5.0) | 0.01 |
| ≥ 3 |  | 55 (14.3) | 50 (2.6) | 0.43 | 101 (5.0) | 87 (3.9) | 0.05 |
| **Comorbidities** |  |  |  |  |  |  |  |
| Hypertension | 2,307 | 283 (73.7) | 1,164 (60.5) | 0.28 | 1,341 (66.5) | 1,392 (62.7) | 0.08 |
| Diabetes | 2,307 | 188 (49.0) | 767 (39.9) | 0.18 | 1,020 (50.6) | 919 (41.4) | 0.19 |
| Dyslipidemia | 2,307 | 205 (53.4) | 825 (42.9) | 0.21 | 813 (40.4) | 988 (44.5) | -0.08 |
| Atrial fibrillation | 2,307 | 116 (30.2) | 551 (28.7) | 0.03 | 665 (33.0) | 645 (29.1) | 0.08 |
| Myocardial infarction | 2,307 | 78 (20.3) | 144 (7.5) | 0.38 | 322 (16.0) | 220 (9.9) | 0.18 |
| Stroke | 2,307 | 51 (13.3) | 160 (8.3) | 0.16 | 208 (10.3) | 198 (8.9) | 0.05 |
| Coronary artery disease | 2,307 | 113 (29.4) | 514 (26.7) | 0.06 | 485 (24.1) | 617 (27.8) | -0.09 |
| Chronic obstructive pulmonary disease | 2,307 | 84 (21.9) | 213 (11.1) | 0.29 | 280 (13.9) | 289 (13.0) | 0.03 |
| **Baseline echocardiography** |  |  |  |  |  |  |  |
| LVEF, % | 2,211 | 30.2 ± 11.5 | 28.9 ± 7.2 | 0.14 | 29.0 ± 9.3 | 29.0 ± 7.0 | <0.01 |
| LVEDD, mm | 2,206 | 61.9 ± 9.4 | 58.8 ± 8.6 | 0.35 | 59.9 ± 9.4 | 59.2 ± 8.4 | 0.08 |
| LVESD, mm | 2,205 | 52.2 ± 10.6 | 50.0 ± 8.6 | 0.22 | 50.7 ± 10.0 | 50.2 ± 8.5 | 0.06 |
| LA, mm | 2,200 | 45.3 ± 8.2 | 44.1 ± 8.3 | 0.14 | 44.6 ± 7.7 | 44.2 ± 8.2 | 0.05 |
| MR severity | 2,307 |  |  |  |  |  |  |
| Trivial/None |  | 32 (8.3) | 201 (10.5) | -0.07 | 251 (12.5) | 224 (10.1) | 0.08 |
| Mild |  | 178 (46.4) | 964 (50.1) | -0.08 | 948 (47.0) | 1,087 (49.0) | -0.04 |
| Moderate |  | 120 (31.3) | 487 (25.3) | 0.13 | 579 (28.7) | 600 (27.0) | 0.04 |
| Severe |  | 45 (11.7) | 162 (8.4) | 0.11 | 211 (10.5) | 195 (8.8) | 0.06 |
| Missing |  | 9 (2.3) | 109 (5.7) | -0.17 | 26 (1.3) | 113 (5.1) | -0.22 |
| **Baseline laboratory data** |  |  |  |  |  |  |  |
| BNP, pg/mL | 1,823 | 1566  [795, 2781] | 1205  [582, 2284] | 0.21 | 1693  [783, 2773] | 1312  [673, 2255] | 0.15 |
| BUN, mg/dL | 2,214 | 33.7 ± 25.2 | 28.6 ± 21.0 | 0.22 | 30.8 ± 20.8 | 28.9 ± 21.0 | 0.09 |
| Serum creatinine, mg/dl ^c^ | 2,123 | 1.8 ± 1.4 | 1.5 ± 1.4 | 0.23 | 1.6 ± 1.1 | 1.6 ± 1.4 | 0.03 |
| eGFR, mL/min/1.73m^2 c^ | 2,123 | 52.1 ± 27.0 | 62.1 ± 31.2 | -0.34 | 58.0 ± 30.1 | 59.1 ± 30.8 | -0.04 |
| Renal function status | 2,307 |  |  |  |  |  |  |
| ≥60 ml/min |  | 132 (34.4) | 887 (46.1) | -0.24 | 759 (37.7) | 973 (43.9) | -0.13 |
| 30–59 ml/min |  | 144 (37.5) | 666 (34.6) | 0.06 | 716 (35.6) | 794 (35.8) | -0.01 |
| <30 ml/min |  | 66 (17.2) | 237 (12.3) | 0.14 | 253 (12.6) | 278 (12.5) | 0.00 |
| Dialysis |  | 42 (10.9) | 133 (6.9) | 0.14 | 287 (14.2) | 173 (7.8) | 0.21 |
| Sodium (Na), mEq/L | 2,300 | 136.9 ± 4.8 | 137.4 ± 4.5 | -0.10 | 137.5 ± 4.2 | 137.4 ± 4.6 | 0.02 |
| Potassium (K), mEq/L | 2,300 | 4.1 ± 0.7 | 4.0 ± 0.6 | 0.15 | 4.1 ± 0.7 | 4.0 ± 0.6 | 0.16 |
| Hemoglobin, g/dL | 2,303 | 12.6 ± 2.5 | 12.9 ± 2.6 | -0.14 | 12.9 ± 2.4 | 12.9 ± 2.6 | -0.02 |
| **Hypoglycemic medications** |  |  |  |  |  |  |  |
| Thiazolidinedione | 2,307 | 3 (0.8) | 17 (0.9) | -0.01 | 7 (0.3) | 18 (0.8) | -0.06 |
| GLP1RA | 2,307 | 1 (0.3) | 2 (0.1) | 0.04 | 4 (0.2) | 3 (0.2) | 0.01 |
| SGLT2i | 2,307 | 36 (9.4) | 108 (5.6) | 0.14 | 134 (6.7) | 135 (6.1) | 0.02 |
| **Other medications in the previous 3 months** |  |  |  |  |  |  |  |
| Beta-blockers | 2,307 | 333 (86.7) | 1,705 (88.7) | -0.06 | 1,729 (85.8) | 1,970 (88.8) | -0.09 |
| MRAs | 2,307 | 268 (69.8) | 1,049 (54.6) | 0.32 | 1,150 (57.1) | 1,255 (56.5) | 0.01 |
| Ivabradine | 2,307 | 118 (30.7) | 309 (16.1) | 0.35 | 372 (18.5) | 402 (18.1) | <0.01 |
| Loop diuretics | 2,307 | 335 (87.2) | 1,644 (85.5) | 0.05 | 1,764 (87.5) | 1,915 (86.3) | 0.04 |
| Digoxin | 2,307 | 97 (25.3) | 380 (19.8) | 0.13 | 336 (16.7) | 457 (20.6) | -0.10 |
| Amiodarone | 2,307 | 83 (21.6) | 250 (13.0) | 0.23 | 430 (21.3) | 327 (14.7) | 0.17 |
| **Other treatments** |  |  |  |  |  |  |  |
| Implantable cardioverter-defibrillator | 2,307 | 22 (5.7) | 37 (1.9) | 0.20 | 74 (3.7) | 67 (3.0) | 0.04 |
| CRT | 2,307 | 11 (2.9) | 6 (0.3) | 0.21 | 21 (1.1) | 18 (0.8) | 0.03 |
| **In-hospital event** |  |  |  |  |  |  |  |
| Hospital days | 2,307 | 17.4 ± 20.1 | 13.6 ± 14.6 | 0.22 | 15.4 ± 14.4 | 14.0 ± 16.2 | 0.09 |
| ICU days | 2,307 | 3.1 ± 8.5 | 2.2 ± 5.3 | 0.12 | 2.3 ± 6.4 | 2.3 ± 5.6 | -0.01 |
| Inotropes | 2,307 | 82 (21.4) | 328 (17.1) | 0.11 | 460 (22.8) | 401 (18.1) | 0.12 |
| Intubation | 2,307 | 21 (5.5) | 60 (3.1) | 0.12 | 81 (4.0) | 77 (3.5) | 0.03 |
| Acute myocardial infarction | 2,307 | 38 (9.9) | 319 (16.6) | -0.20 | 256 (12.7) | 355 (16.0) | -0.09 |
| PCI | 2,307 | 54 (14.1) | 307 (16.0) | -0.05 | 383 (19.0) | 353 (15.9) | 0.08 |

Abbreviations: ACEI/ARB, angiotensin-converting enzyme inhibitor/angiotensin receptor blocker; HF, heart failure; LVEF, left ventricular ejection fraction; LVEDD, left ventricular end-diastolic diameter; LVESD, left ventricular end-systole diameter; LA, left atrial; MR, mitral regurgitation; BNP, B-type natriuretic peptide; BUN, blood urine nitrogen; eGFR, estimated glomerular filtration rate; GLP1RA, glucagon-like peptide-1 receptor agonist; SGLT2i, sodium glucose cotransporter 2 inhibitor; MRA, mineralocorticoid antagonist; CRT, cardiac resynchronization therapy ; ICU, intensive care unit; PCI, percutaneous coronary intervention.

^a^ All covariates listed were used to calculate the propensity score. Values are presented as n (%).

^b^ An absolute standardized difference of < 0.1 indicated a negligible difference, and a value between 0.1 and 0.2 is considered as a small difference.

^c^ Patients with dialysis at baseline were excluded.

**eTable 3. Effectiveness clinical outcomes at the end of follow-up in the new user analysis**

| Outcome | **Data after IPTW^a^** | | | |
| --- | --- | --- | --- | --- |
|  | **Sacubitril-valsartan** | **ACEI/ARB** | **HR or SHR for**  **Sacubitril-valsartan**  **(95% CI) ^b^** | ***P* value** |
| Composite of rehospitalization for HF and death | 514 (25.5) | 853 (38.4) | 0.77 (0.54–1.11) | 0.165 |
| Death | 90 (4.5) | 229 (10.3) | 0.52 (0.27–0.99) | 0.049 |
| Rehospitalization for HF | 484 (24.0) | 759 (34.2) | 0.84 (0.74–0.94) | 0.004 |

Abbreviations: ACEI/ARB, angiotensin-converting enzyme inhibitor/angiotensin receptor blocker; CI, confidence interval; HF, heart failure; HR, hazard ratio; IPTW, inverse probability of treatment weighting; SHR, subdistribution hazard ratio.

^a^ Values are presented as n (%).

^b^ Additionally adjusted for body mass index, diabetes, myocardial infarction, mitral regurgitation severity, B-type natriuretic peptide, renal function status, potassium, amiodarone, inotropic agents, which were the variable with SMD>0.1 after IPTW in incidence user analysis.

**eTable 4. Effectiveness and safety clinical outcomes in multivariable adjustment analysis**

| Outcome | Sacubitril-valsartan  (n = 384) | ACEI/ARB  (n = 3,352) | HR or SHR for  Sacubitril-valsartan  (95% CI) | *P* value |
| --- | --- | --- | --- | --- |
| **Effectiveness outcomes at the end of study** |  |  |  |  |
| Composite of rehospitalization for HF and death | 123 (32.0) | 1,391 (41.5) | 0.83 (0.66–1.04) | 0.111 |
| All-cause death | 26 (6.8) | 370 (11.0) | 0.62 (0.37–1.05) | 0.075 |
| Rehospitalization for HF | 113 (29.4) | 1,254 (37.4) | 0.90 (0.71–1.14) | 0.384 |
| Non-fatal myocardial infarction | 5 (1.3) | 109 (3.3) | 0.83 (0.31–2.21) | 0.706 |
| Non-fatal ischemic stroke | 2 (0.52) | 69 (2.1) | 0.37 (0.04–3.19) | 0.363 |
| **Safety outcomes ^a^** |  |  |  |  |
| Worsening renal function ^b^ | 90 (23.4) | 880 (26.3) | 1.13 (0.84–1.53) | 0.413 |
| Composite of decline of eGFR >50% or progression to ESRD ^a^ | 62 (16.1) | 719 (21.4) | 0.77 (0.50–1.18) | 0.227 |
| Decline of eGFR >50% from baseline | 35 (9.1) | 484 (14.4) | 0.85 (0.54–1.33) | 0.473 |
| Progression to ESRD | 37 (9.6) | 320 (9.5) | 0.68 (0.28–1.69) | 0.411 |
| Creatinine ≥2.5 mg/dL | 83 (21.6) | 760 (22.7) | 0.86 (0.60–1.24) | 0.432 |
| Creatinine ≥3 mg/dL | 68 (17.7) | 610 (18.2) | 0.96 (0.62–1.47) | 0.845 |
| Kalium ≥6 mg/dL | 22 (5.7) | 228 (6.8) | 0.87 (0.44–1.73) | 0.690 |

Abbreviations: ACEI/ARB, angiotensin-converting enzyme inhibitor/angiotensin receptor blocker; CI, confidence interval; eGFR, estimated glomerular filtration rate; ESRD, end-stage renal disease, HF, heart failure; HR, hazard ratio; IPTW, inverse probability of treatment weighting; SHR, subdistribution hazard ratio.

^a^ Patients with dialysis at baseline were excluded.

^b^ An increase of creatinine more than 0.5 and a decrease of eGFR more than 25%.

**eTable 5. The daily prescribed dose of sacubitril–valsartan at baseline, 3 months, 12 months, and end of follow-up.**

|  | **Sacubitril-valsartan** | | | |
| --- | --- | --- | --- | --- |
|  | **Baseline** | **3 months** | **12 months** | **End of follow-up** |
| Available patient number | 375* | 277 | 270 | 270 |
| Dose per day |  |  |  |  |
| 25mg | 3 | 2 | 2 | 3 |
| 50mg | 35 | 20 | 17 | 16 |
| 100mg | 200 | 128 | 110 | 107 |
| 200mg | 110 | 101 | 97 | 94 |
| 400mg | 27 | 26 | 44 | 50 |
| Mean dosage, mg | 145.7 ± 87.3 | 160.5 ± 93.2 | 181.1 ± 109.2 | 186.6 ± 113.6 |
| Drug cessation (%) # | - | 98 (26.1) | 105 (28.0) | 105 (28.0) |

* A total of 384 patients were included, but 9 had unknown drug frequency at baseline.

# Due to those who were lost to follow-up and those who died during follow-up.

**eFigure 1. Safety outcomes by renal function subgroups in the IPTW cohort.**

IPTW, inverse probability of treatment weighting; ACEI/ARB, angiotensin-converting enzyme inhibitor/angiotensin receptor blocker; SHR, subdistribution hazard ratio; CI, confidence interval; eGFR, estimated glomerular filtration rate, ESRD, end-stage renal disease.

^a^ Patients receiving dialysis at baseline were excluded.


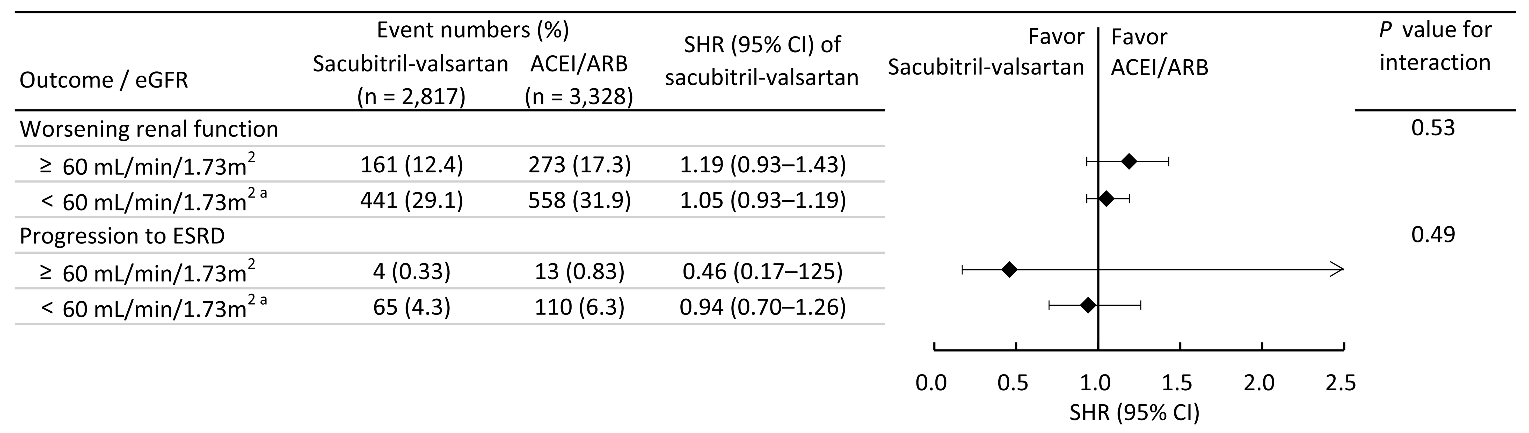


**eFigure 2. Level of NT-pro BNP at baseline and 12 months of follow-up in the IPTW cohort.**

NT-pro BNP, N-terminal pro-B type natriuretic peptide.

**
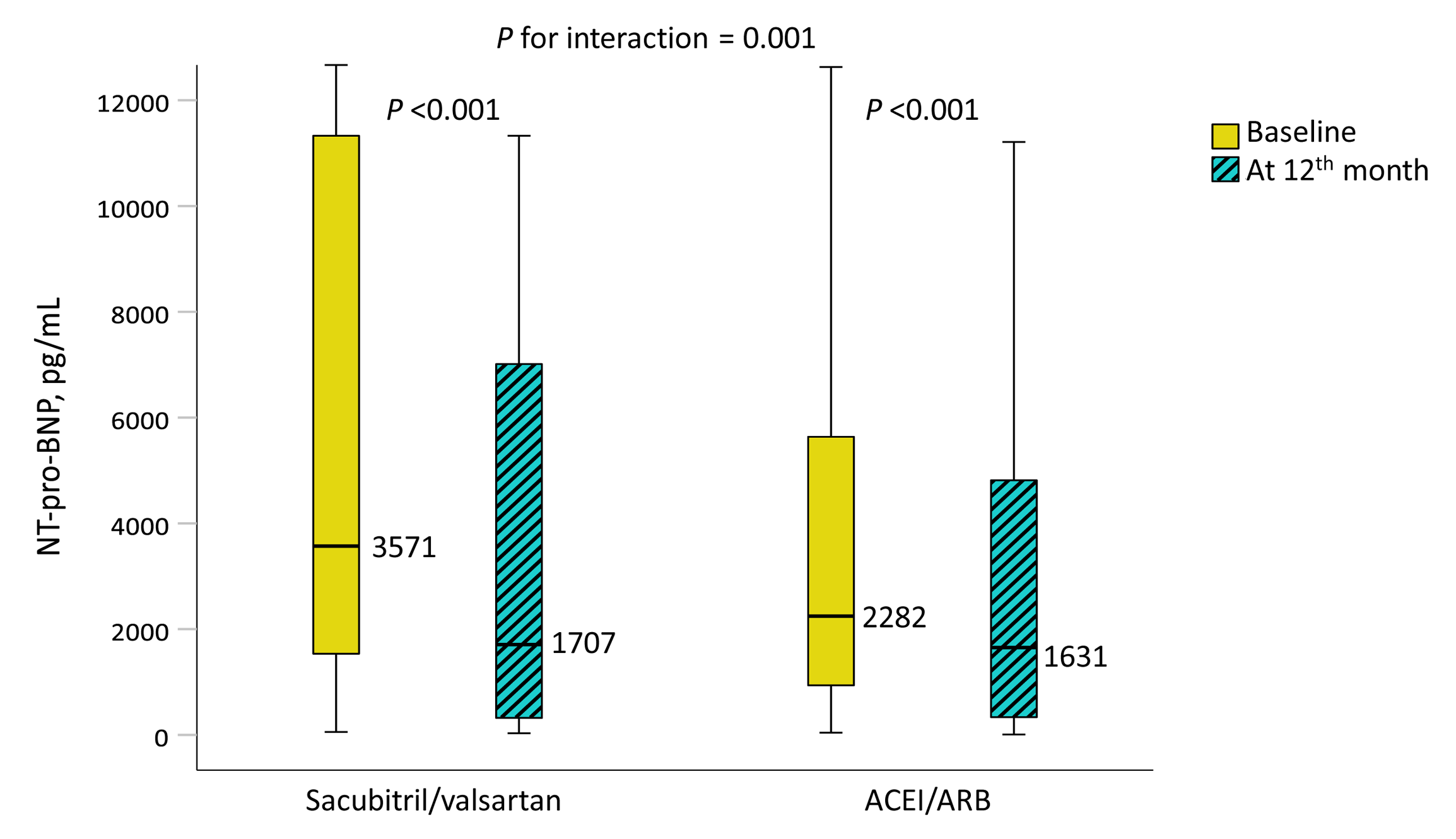
**

**eFigure 3. Cumulative incidence of rehospitalization for heart failure and death by ACEI or ARB subtype in the original cohort.**

ACEI, angiotensin-converting enzyme inhibitor; ARB, angiotensin receptor blocker.


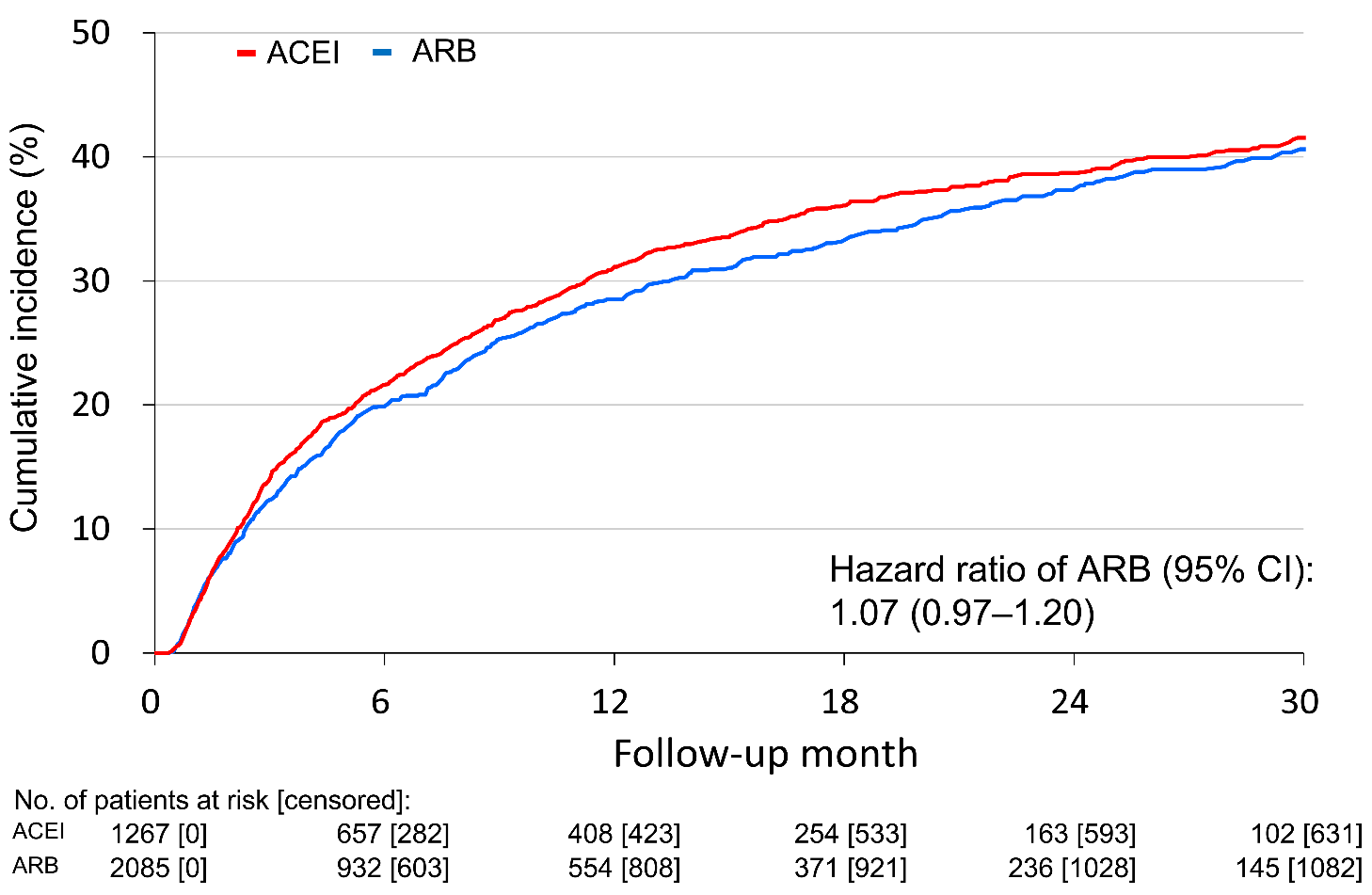

Supplement: Supplementary file 2 [file mmc2.docx]
